# Spontaneous perforation of Meckel's diverticulum: a case report and review of literature

**DOI:** 10.11604/pamj.2015.20.319.5980

**Published:** 2015-04-01

**Authors:** Robleh Hassan Farah, Prude Avala, Driss Khaiz, Fatmazahra Bensardi, Khalid Elhattabi, Rachid Lefriyekh, Saad Berrada, Abdelaziz Fadil, Najib Ouariti Zerouali

**Affiliations:** 1 Service des Urgences de Chirurgie Viscérale, Pavillon 35, Centre Hospitalier Universitaire Ibn Rochd, Casablanca, Morocco

**Keywords:** Meckel′s diverticulum (MD), Perforation, Acute appendicitis, Peritonitis

## Abstract

Meckel's diverticulum is the commonest congenital abnormality of the gastrointestinal tract. Hemorrhage, obstruction and inflammation are the three main categories of complications resulting from Meckel's diverticulum. Spontaneously perforation of Meckel's diverticulum is very rare and mimics acute appendicitis. We report a case of 26 year-old male, who presented since 5 days worsening abdominal pain predominantly in the right iliac fossa associated with high grade fever. On physical examination his abdomen was distended with guarding and rigidity. A provisional diagnosis of appendiculaire peritonitis was made. Our patient had an emergency laparotomy, where a perforated Meckel's diverticulum and advanced peritonitis were discovered. A diverticulectomy with ileostomy were performed. Heterotopic mucosa of diverticulitis was confirmed on histopathology. The patient made an uneventful recovery postoperatively and ileostomy reconstruction was done two months later. This case report is an interesting and unusual case of Meckel's diverticulum complications and highlights the importance of considering Meckel's diverticulum as a differential diagnosis in every patient presenting with acute abdomen.

## Introduction

Meckel's diverticulum (MD), first described in 1808, results from failure of complete obliteration of the vitelline duct. It is a common anomaly of the small intestine that occurs in approximately 2% of the population, often found incidentally at the time of abdominal exploration. The complications associated with MD include inflammation, perforation, hemorrhage, intussusception, volvulus, intestinal obstruction, and malignant transformation. The total lifetime complication rate has been reported to be around 4% [1]. MD is the most prevalent congenital anomaly of the gastrointestinal tract, affecting approximately 2% of the general population. A 3:2 male to female ratio has been reported. Meckel's diverticula are designated true diverticula because their walls contain all of the layers found in normal intestine. Their location varies among individual patients, but they are usually found in the ileum within 100cm of the ileocecal valve. Approximately 60% of Meckel's diverticula contain heterotopic mucosa, of which over 60% consist of gastric mucosa [2–4]. Other pancreatic mucosa (5%) and less commonly colonic mucosa, endometriosis, hepatobiliary tissue, which are responsible for other complications like hemorrhage, chronic peptic ulceration and perforation. Majority of the meckel′s diverticulum remain silent and are diagnosed incidentally during small bowel contrast study, laparoscopy or laparotomy done for unrelated conditions, or until complications arise from the diverticulum [3]. A commonly quoted “rule of 2s”also applies: 2% of the population has the anomaly, it is approximately 2 inches in length, it is usually found within 2 feet of the ileocecal valve, it is often found in children less than 2 years of age and it affects males twice as often as females. Although these are good general guidelines, they are not based on accurate data [4]. The overall lifetime complication rate is approximately 4%. The most common presentation associated with symptomatic Meckel's diverticula is bleeding, followed by intestinal obstruction, diverticulitis, intussusceptions and neoplasm [2]. Here we provide an illustrative presentation, outlining one of the rare complications Of Meckel's diverticulum in adults. Case presentation

## Patient and observation

**Figure 1:**
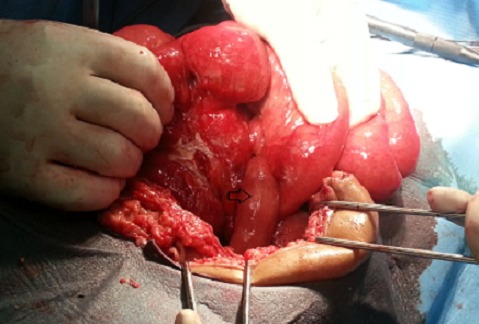
Peroperative view of the perforated the Meckel's diverticulum before excision.

**Figure 2:**
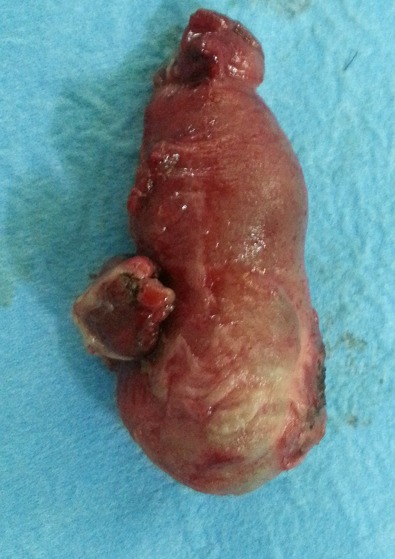
Anatomical specimen after excision

A 26-year-old male, who presented 5 days ago, a history worsening abdominal pain associated with fever and vomiting. On his admission, patient was toxic, but his vital signs were within normal range. A physical examination demonstrated abdomen was distended with guarding and rigidity. His blood analysis revealed slight elevated blood count, his white blood cells (WBC) were 11,220/µl (normal values 4.6 to 10.2 × 103/mL) and 74% of them were neutrophils (normal values 40 to 75%). The rest of the routine preoperative blood tests and his erect chest and abdominal X-rays were unremarkable. A provisional diagnosis of appendicular peritonitis was made and initial management included intravenous fluid resuscitation and antibiotic coverage. No other examinations were performed and, after our patient gave his written consent, he was taken to the operating theatre and under general anesthesia; a lower umbilical median incision was performed. A normal appearing appendix was identified, which did not have any remarkable sign of inflammation that could explain the contraction and the peritoneal irritation. During the operation, some serous peritoneal fluid was observed between the small intestine loops and Douglas pouch. An examination of the small bowel revealed an inflamed and perforated MD at 75cm proximal to the ileocecal valve (Figure 1). The Meckel's diverticulum was perforated at its base (Figure 2). A Meckel's diverticulectomy & ileostomy were performed. Abundant peritoneal toilet was done with normal saline solution and drainage of douglas pouche by salem's tube. Heterotopic mucosa of diverticulitis was confirmed on histopathology. The patient made an uneventful recovery postoperatively and ileostomy reconstruction was done two months later. Clinical follow up over the next one year was unremarkable

## Discussion

Meckel's diverticulum is a congenital anomaly found in approximately 2% of the general population. Complications develop in only 4% of patients with this malformation, with most cases presenting in childhood [2]. Complications of Meckel's diverticulum include hemorrhage, bowel obstruction, inflammation, perforation, intussusception, volvulus and malignant transformation. The pre-operative diagnosis of a patient with Meckel's diverticulum often presents a challenge to the clinician in both children and adults, because presenting symptoms can be non-specific and the differential diagnosis broad [5]. We report a complicated and unusual case of a patient with a spontaneous perforated Meckel's diverticulum who presented with acute abdomen. The patient required an open laparotomy for definitive diagnosis and management. Complications in patients with Meckel's diverticulum are rare; most patients remain asymptomatic for life [6]. The perforation of a Meckel's diverticulum may mimic acute appendicitis and present as an acute abdomen [7]. The perforation of a Meckle's diverticulum is either caused by; foreign body due to irritation of foreign body and pressure necrosis of the diverticulum wall, or spontaneous perforation due to progressive inflammation of Meckle's diverticulum wall as our case which produced peritonitis. Rarely, cases of perforation following blunt abdominal trauma have been reported, the first being by Park and Lucas in 1970. Four such cases have been reported in the medical literature. Ekwunife et al report the first from Africa [8]. A preoperative diagnosis of a complicated MD may be challenging because of the overlapping clinical and imaging features of other acute surgical and inflammatory conditions of the abdomen. A more specific diagnosis, however, will lead to greater recourse to a laparoscopic approach in its treatment [9].

## Conclusion

Meckel's diverticulum complications are uncommon and challenge to diagnose. Early diagnosis and timely operative intervention must occur in order to provide the best outcome for these patients. Spontaneous perforated MD often presents as acute abdomen and its preoperative diagnosis is difficult. To patients with sudden abdominal pain mimic acute appendicitis accompanied by a past medical history of bloody stools and/or chronic recurrent abdominal pain, perforated MD should be kept in mind as a differential diagnosis.
